# Clinical outcome of a headache-specific multidisciplinary treatment program and adherence to treatment recommendations in a tertiary headache center: an observational study

**DOI:** 10.1007/s10194-011-0348-y

**Published:** 2011-05-05

**Authors:** Charly Gaul, Christina van Doorn, Nadine Webering, Martha Dlugaj, Zaza Katsarava, Hans-Christoph Diener, Günther Fritsche

**Affiliations:** Headache Center, Department of Neurology, University Hospital Essen, University Duisburg-Essen, Hufelandstraße 55, 45147 Essen, Germany

**Keywords:** Migraine, Headache, Multidisciplinary treatment program, Adherence

## Abstract

This study investigated the outcome of a 5-day headache-specific multidisciplinary treatment program (MTP) and the adherence to treatment recommendations in 295 prospectively recruited consecutive headache patients [210 migraine, 17 tension-type headache (TTH), 68 combination headache, including 56 medication-overuse headache (MOH)]. Headache frequency decreased from 13.4 (±8.8) to 8.8 (±8.0) days per month after 12–18 months. Forty-three percent of the participants fulfilled the primary outcome (reduction of headache frequency of ≥50%), which was less likely in patients with combination of migraine and TTH compared to migraine (OR = 3.136, *p* = 0.002) or TTH (OR = 1.029, n.s.). Increasing number of headache days per month (OR = 1.092, *p* ≤ 0.0001) and adherence to lifestyle modifications (OR = 1.269, *p* = 0.004) predicted primary outcome. 51 of 56 MOH patients were treated successfully. Thirty-five percent of the patients were adherent to pharmacological prophylaxis, 61% to relaxation therapy, and 72% to aerobic endurance sports. MTP is effective in headache treatment. Adherence to therapy was associated with better outcome.

## Introduction

Primary headaches, in particular migraine and tension-type headache (TTH) cause severe burden of disease and create high costs in the health care system [1, 2]. Increasing headache frequency in migraine or tension-type headache leads to chronic headache, which is by definition headache on more than 15 days per month [3]. Frequent headache increases the risk of frequent intake of triptans or analgesics, which may result in medication-overuse headache (MOH) [3]. Usually MOH is treated by withdrawal from the overused drugs [4]. Longitudinal studies in academic headache clinics reported relapse rates from 30 to 41% 1 year after initial outpatient withdrawal treatment in MOH [5–8].

Treatment of chronic migraine, TTH, and MOH is challenging for general practitioners and neurologists in private practice. Providing patients with frequent or severe headaches with information on the pathophysiology and treatment is time-consuming and cannot be provided in a setting with a very limited time budget per patient. Therefore, multidisciplinary treatment approaches (MTP) were established during the past decade in a few academic headache centers around the world [9, 10]. Education about acute and prophylactic treatment is needed and may improve adherence to pharmacological as well as to non-pharmacological therapy. The term adherence is used to describe an active role and collaborative involvement of the patient in the implementation of a therapeutic regime. Compliance refers to the degree to which patients are obedient to medical treatment recommendations more generally [11]. Noncompliance and non-adherence are well-known problems of therapy and potentially result in treatment failure. Little is known about adherence to prophylactic headache treatment. A recent study reports non-adherence to medical prophylaxis in headache therapy in up to 35% of the patients. Remarkably, neither demographic characteristics nor any of the disease-specific variables were significantly associated with adherence [12]. Increasing adherence may result in improved clinical outcome. Therefore, as a first step, we undertook a study to investigate adherence in patients with difficult-to-treat headaches and its influence on headache frequency. We hypothesized that integrated headache care would lead to reasonable adherence to treatment recommendations and satisfying clinical outcome.

## Methods

This is a prospective observational study evaluating the outcome of headache patients following the MTP of the West German Headache Center in the year 2008. Informed consent was obtained from all patients. The study was approved by the local ethics committee. Inclusion criteria were (a) ≥16 years at the time of MTP and (b) diagnosed as migraine, TTH (or both) and/or MOH, and (c) adequate knowledge of German language.

All study participants were seen in the outpatient headache center prior to MTP by a neurologist who diagnosed the headache type according to the ICHD-II criteria (Headache Classification Subcommittee 2004) and collected information about demography and frequency and intake of acute and preventive drugs. Patients were referred to the 5-day MTP in case of frequent headache (migraine, TTH, or both), self-reported high burden of disease and when MOH was diagnosed.

### Setting and concept of the MTP

The West German Headache Center is part of the outpatient service of the Department of Neurology at the University Hospital Essen. The center provides medical care for patients with hard-to-treat headaches referred by neurologists and headache specialists or their insurance company. The multidisciplinary team comprises neurologists, behavioral psychologists, physical and sports therapists, headache nurses and consultants from psychosomatic medicine, psychiatry and dentistry if needed. The majority of patients is referred back with a treatment concept, while approximately 10% of the patients are referred to the outpatient multidisciplinary day clinic treatment program (MTP). Participation in MTP was recommended to patients suffering from high headache frequency, high individual burden of disease, diagnosed as MOH or difficult to treat primary headaches. Furthermore, possibility of participation depends on reimbursement of treatment costs by the health insurance and the willingness of the patient to participate. The MTP focusing on education and treatment is performed in groups of ten patients visiting the Headache Center for 5 days in a row. Each day starts with a 60-min lecture on headache education by a physician. These lessons focus on informing patients about the symptoms, etiology and pathophysiology of headaches, treatment options and efficacy and possible adverse events of acute and prophylactic medication and correct use of these medications. This is followed by group sessions with a behavioral psychologist for 90 min everyday. Psychological group sessions provide behavioral recommendations and discussions about lifestyle, individual concepts of headache and its etiology, headache triggers and patients’ attitude towards them and idea of health and sickness. In addition, patients have the opportunity to exchange individual experiences and opinions and learn not only from the professional staff but also from each other [13]. Important goals are individual identification and modification of trigger factors, stress management and prevention or at least reduction in the number of headache episodes through pharmacological and psychological interventions. Psychological training tries to implement different recommendations on lifestyle modification: (1) do not to exceed 10 medication intake days per month; (2) accept the headache and do not rebel against it; (3) incorporate more breaks in daily life and avoid hecticness; (4) pay more attention to your own needs; (5) establish regular sleep times all nights in the week; (6) establish regular mealtimes all days in the week; (7) change more slowly between tension and relaxation; (8) pay less attention to potential trigger factors.

Patients perform 60 min of relaxation training (PMR) led by a psychologist, followed by physical therapy (60 min) and aerobe ergometer training (60 min) under guidance of a professional physiotherapist in the afternoon. During the week, these exercises are performed everyday by all patients. Subsequently, patients have the opportunity to test various forms of endurance training (mainly using sports gym equipment) and find out which one is suitable for them.

In addition to the group sessions, each patient has two face-to-face appointments with a neurologist (2 × 30 min) and a psychologist (1 × 60 min). These appointments focus on individual medical therapy and individual psychosocial background or psychiatric comorbidity.

### Study procedures

The questionnaire-based follow-up telephone interviews were performed 12–18 months (some phone calls had to be delayed due to poor reachability of some patients) after MTP by a trained medical student. Patients were interrogated about adherence to treatment recommendations, reasons for non-adherence, and headache and medication intake days. The amount of headache days per month was reported by the patients partially on the basis of their headache diaries. A reduction in headache frequency (days per month) of ≥50% was defined as the primary outcome. This endpoint was suggested by the IHS [14]. Reduction of headache days per month, frequency of medication intake to treat headache attacks, adherence to treatment recommendations, and long-term success in MOH as well as newly occurring MOH were secondary outcome parameters.

### Statistics

The data gained prior to and after MTP were entered into a database by double entry and quality checked. Continuous variables were compared utilizing the Student *t* test or the Mann–Whitney *U* test when variables were not normally distributed. χ^2^ tests were used for comparison of categorical variables. *p* values below 0.05 (two-tailed) were defined as significant. Binary logistic regression and ANOVA were computed to determine predictors influencing primary outcome, which was defined as a reduction of ≥50% in headache days per month resulting in odds ratios (OR) with 95% confidence intervals (CI). We used a stepwise selection procedure to identify the most important predictors of positive outcome using a *p* value of 0.05 as inclusion or exclusion criteria for the logistic regression. Furthermore, we decided to compute a second model which was adjusted for age, gender, number of days with intake of acute medication per month, adherence to pharmacological prophylaxis, PMR, and aerobic endurance sports as potential confounders. Additionally, we computed a binary logistic regression with the variable headache days per month at baseline stratified in six groups (1–5, 6–10, 11–15, 16–20, 21–25, and 26–30 days) to identify the number of headache days which predicts most for primary outcome. All statistical analyses were performed with PASW Statistics 18.0.0.

## Results

### Patient characteristics

In 2008, 3,229 de novo patients were treated in the headache center, of whom 362 (11.2%) visited the MTP, which was the intended population for this follow-up study. Thirty-eight patients were lost to follow-up, and 29 were excluded due to unwillingness to participate (*n* = 2), headache diagnoses other than migraine, TTH or MOH (*n* = 10), age under 16 years (*n* = 8), lacking knowledge of German language (*n* = 6) and early termination of the MTP (*n* = 3). The demographic and the diagnostic characteristics of the included 295 patients are displayed in Table 1. The excluded 67 patients were characterized and differed significantly in age (drop-outs are on average 4 years younger) and had on average 4 years shorter duration of headache, whereas no difference was found in all other baseline data.

**Table 1:** Demographic and diagnostic characteristics of the study sample

Demography	
*N*	295
Age (years)	41 ± 12.9
Range	16–76
Gender	
Men (%)	33 (11)
Women (%)	262 (89)
Clinical features	
Diagnosis	
TTH and migraine (%)	68 (23)
Migraine (%)	210 (71)
Without aura (%)^a^	217 (78)
With aura (%)^a^	61 (22)
Episodic (%)^a^	217 (78)
Chronic (%)^a^	61 (22)
TTH (%)	17 (6)
Frequent episodic (%)	41 (48)
Chronic (%)	44 (52)
MOH^b^ (%)	56 (19)
Other headache diagnoses (%)	0 (0)
Headache duration (years)	
All, mean (range)	19 (1–62)
TTH, mean (range)	15 (1–50)
Migraine, mean (range)	19 (1–62)
Allocation of headache duration^c^	
<5 years (%)	27 (9)
5–10 years (%)	76 (26)
>10 years (%)	186 (63)
Headache frequency (days per month), mean (range)	13 (1–30)
Intake frequency of drugs for acute headache episodes (days per month), mean (range)	9 (0–30)

*TTH* tension-type headache, *MOH* medication overuse headache

^a^Patients suffering from migraine and combination of TTH and migraine

^b^Multiple diagnoses possible

^c^Not available for all patients

The analysis of headache characteristics revealed a significantly (*p* < 0.001) higher headache frequency in TTH than in migraine patients, whereas the patients suffering from migraine and TTH showed headache frequencies which were in between the two single entities (Table 2). In a further analysis, patients with additional MOH had the highest headache frequency (mean 22 days) and reported analgesic or triptan intake on average on 22 days per month.

**Table 2:** Headache characteristics of the included patients

Primary headache diagnosis	Gender (M/F)	Age Mean years (range)	Headache Duration (years, range)	Headache frequency Days/month (range)	Analgesic/triptan intake frequency Days/month (range)	MOH (%)
TTH (*n* = 17)	7/10	40 (18–67)	13 (1–30)	27 (4–30)	8 (0–30)	3 (18)
Migraine (*n* = 210)	19/191	42 (16–76)	19 (1–62)	11 (1–30)	9 (0–30)	29 (14)
TTH & Migraine (*n* = 68)	7/61	40 (17–70)	20 (1–50)	18 (2–30)	10 (0–30)	12 (18)

*TTH* tension-type headache, *MOH* medication overuse headache

### Outcome

The primary endpoint after MTP, defined as a reduction in headache frequency ≥50% at follow-up, was observed in 127/295 patients (43%) and differs between the different groups of primary headaches. The highest amount of patients (53%) who reached primary outcome criterion is found in TTH patients compared to 45% in migraine patients and 29% in patients who suffered from both migraine and TTH.

**Fig. 1:**
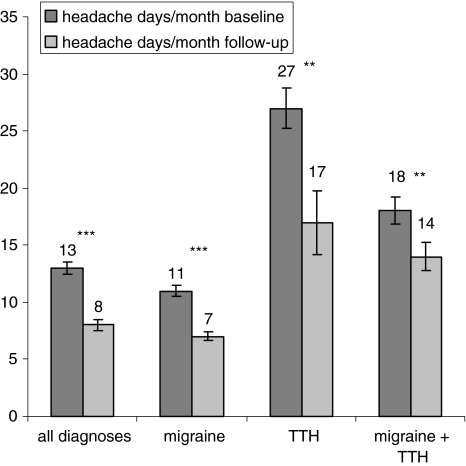
Change in number of headache days per month at baseline and follow-up. Headache days per month (mean ± standard error of mean) at baseline and at follow-up after 12–18 months. *p < 0.05; **p < 0.01; ***p < 0.001 (comparison by *t* tests). *TTH* tension-type headache

Headache frequency decreased in all patients by 5 days per month on average. Concerning the absolute reduction of headache days, patients with TTH improved most (mean: −10 days per month; *p* = 0.010) compared to patients with migraine (mean: −4 days per month; *p* < 0.0001), and those with migraine and TTH (mean: −5 days per month; *p* = 0.009) (Fig. 1). However, patients with TTH were less likely to reach the primary endpoint because of the high number of 27 headache days at baseline. Thirty-five patients (12%) reported no change in headache frequency, while 69 patients (23%) showed an increase in headache days. Changes in headache frequency are shown in Fig. 1.

**Fig. 2:**
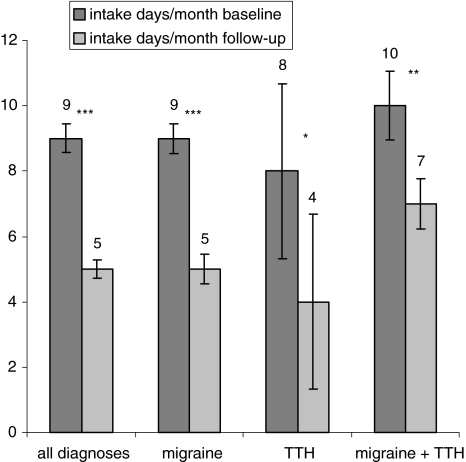
Change in acute medication intake frequency at baseline and follow-up. Intake days of acute medication (mean ± standard error of mean) at baseline and at follow-up after 12–18 months. *p < 0.05; **p < 0.01; ***p < 0.001 (comparisons of means by *t* tests). *TTH* tension-type headache

In parallel with the reduction of headache days, a reduction of days with intake of acute medication (analgesics or triptans) from 9 to 5 days per month (*p* < 0.0001) could be observed (Fig. 2).

### Predictors for primary outcome

The binary logistic regression analysis revealed that the number of headache days per month at baseline (OR = 1.092, 95% CI = 1.034–1.144; *p* < 0.0001) and the number of implemented lifestyle recommendations (OR = 1.269, 95% CI = 1.079–1.492; *p* = 0.002) were significant predictors for primary outcome. Compared to patients with the combination of migraine and TTH, patients with migraine were more likely to fulfill primary outcome (OR = 3.136, 95% CI = 1.517–6.483; *p* = 0.002) as were patients with TTH (OR = 1.029, 95% CI = 0.275–3.849; n.s.). The detailed results of this analysis are shown in Table 3.

**Table 3:** Association of primary outcome of MTP and headache diagnosis, headache days before training and implementation of lifestyle recommendations

Covariates	Unadjusted OR (95% CI)	*p* value	Adjusted OR (95% CI)	*p* value
Primary headache diagnosis				
Migraine	2.867 (1.437–5.719)	**0.003**	3.136 (1.517–6.483)	**0.002**
TTH	1.303 (0.404–4.206)	0.658	1.029 (0.275–3.849)	0.966
Both	1.00 (reference)		1.00 (reference)	
Headache days per month before MTP	1.076 (1.039–1.114)	**<0.0001**	1.092 (1.043–1.144)	**<0.0001**
Number of implemented lifestyle recommendations	1.272 (1.087–1.488)	**0.003**	1.269 (1.079–1.492)	**0.004**

Odds ratios (95% CI) of primary outcome (≥50% reduction in headache frequency) were computed using logistic regressions adjusted for age, gender, years of suffering from headache, adherence to pharmacological prophylaxis, adherence to PMR, adherence to aerobic endurance sports, and intake days of acute medication. Significant *p* values are presented in bold. *p* value was <0.0001 for both models

The interval between participation in MTP and follow-up interview was no significant predictor for outcome.

Student *t* tests revealed that patients who fulfill primary outcome had a mean of 16 headache days per month and implemented six or more lifestyle recommendations, while patients who did not improve suffered from only 12 headache days per month and implemented less than six recommendations. Furthermore, we computed a separate binary logistic regression analysis to determine which number of headache days per month predicts primary outcome. We stratified the headache days per month into six groups in 5-day intervals, ranging from 1 to 5 days of headache to 26–30 days. The category of patients with 1–5 headache days per month was determined as the reference category. In general, the odds ratios increased with the number of headache days per month, while patients in the category 21–25 days have the highest chance of reaching the primary outcome (OR = 13.667, 95% CI = 2.553–73.162; *p* = 0.002).

The MOH was diagnosed in 56 patients at admission to MTP of whom 30 overused analgesics, 9 overused triptans and 16 had a combined overuse of analgesics and triptans while only one patient overused opioids. Fifty-one of the 56 patients (91%) showed long-term success. At the time of follow-up, five of these 56 patients still fulfilled criteria for MOH and were therefore classified as treatment failures (persistent MOH or relapse). Another eight patients who had episodic headaches at baseline developed an increase of headache frequency and were overusing headache medication at follow-up. Four patient overused analgesics at follow-up, and eight patients showed a combined overuse of analgesics and triptans, while only one patient overused triptans.

### Adherence to treatment recommendations

#### Medical prophylaxis

Patients were asked about their adherence to therapeutic recommendations. Patients received recommendation on the following prophylactic drugs: topiramate (*n* = 88), betablockers (*n* = 66), magnesium (*n* = 34), amitriptyline (*n* = 30), flunarizine (*n* = 16), tizanidine (*n* = 8), opipramol (*n* = 6), duloxetine (*n* = 5), valproate (*n* = 5), butterbur (*n* = 1), and lamotrigine (*n* = 1). 26 patients take combinations of those medications. Altogether, 244 of 295 patients were advised to take preventive medication, of whom 22 (9%) did not fill the initial prescription at all. Ninety-six patients (39%) followed the recommendation for a limited time (mean 5 months), of whom 13 (5%) received the recommendation to terminate prophylaxis after a mean period of 8 months after MTP. Eighty-six patients (35%) followed the prescription at least until the telephone interview. The remaining 40 patients (16%) changed medication. The reasons for changes or termination are shown in Table 4.

**Table 4:** Reasons for non-adherence to pharmacological prophylaxis

Reason	Number of patients (%^a^)
Ineffectiveness	19 (12.8)
Side effects	41 (27.6)
Fear of side effects	2 (1.4)
Pregnancy	4 (2.7)
Physician’s advice at MTP	13 (8.8)
Denial of daily medication intake	6 (4.1)
General non-compliance	14 (9.5)
Discontinuation	6 (9.5)
No further need because of improvement	9 (6.1)
Preference of non-pharmacological approaches	4 (2.7)
Contraindications	1 (0.7)
Advice of another person (change/termination of prophylaxis)^b^	47 (31.8) (40/7)
Not specified	8 (5.4)

^a^Percentage of patients who did not ingest prescribed prophylaxis until follow-up; multiple answers were possible

^b^Advice of another person: after MTP, e.g., general practitioner, neurologist in private practice, physician in West German Headache Center; main reasons for change or termination were side effects (19%) and ineffectiveness (17%)

#### Progressive muscle relaxation, aerobic endurance sports, and lifestyle recommendations

All patients were instructed in progressive muscle relaxation (PMR) during MTP, of whom 59 patients (20%) never performed PMR during follow-up. Fifty-six (19%) patients executed PMR for 3 months on average, and 180 (61%) patients still performed PMR on a regular basis at the time of the telephone interview. On average, patients practiced PMR on 3 days per week.

Forty-five (15%) patients discontinued aerobic endurance sports after MTP; 38 (13%) patients continued for 6 moths on average, and 212 (72%) patients strictly followed the recommendation. Patients practiced aerobic endurance sports on 3 days per week on average as recommended.

Furthermore, patients received eight recommendations to change their lifestyle. Fifty-six percent of the patients implemented at least six recommendations. Adherence to more than five recommendations was associated with a significant reduction in headache frequency and the primary endpoint of ≥50% reduction of headache days per month. In a multivariate analysis, the strongest predictor for headache reduction was implementation of the lifestyle modification to “accept the headache and do not rebel against it”.

Patients who did not fulfill primary outcome criterion were less adherent to medical prophylaxis (33.3 vs. 35.4%, n.s.), PMR (60.7 vs. 61.4%, n.s.), aerobic endurance sports (69.6 vs. 74.8%, n.s.), and lifestyle modification (48.8 vs. 64.6%, *p* = 0.009) than those patients who showed a reduction of ≥50% in headache days per month.

To determine which combinations of implementation of the various recommendations were predictors for primary outcome, we computed a variance analysis (ANOVA) containing the factors “adherence to pharmacological therapy”, “adherence to PMR”, “adherence to aerobic endurance sports”, and “adherence to lifestyle recommendations”. Most of the combinations including the non-pharmacological measures showed significant effect on primary outcome while the exclusive pharmacological therapy had no significant influence (*p* = 0.943) on the primary endpoint.

Patients were asked which recommendations (multiple answers possible) were considered as most helpful in reducing headache frequency. 57% of the patients regarded the advice to change their daily behavior as the most important advice resulting in improvement of headache.

## Discussion

Our study demonstrates that an outpatient MTP is effective in treating patients with frequent migraine, TTH, and MOH and results in over-all satisfying adherence to relaxation training, aerobic endurance sports, and implementation of lifestyle recommendations. Forty-three percent of all patients reached the primary endpoint of a ≥50% reduction of headache days per month. Therefore, MTP was effective in reducing both headache days (on average −5 days/month) and medication intake (on average −4 days/month). This is notable regarding the fact that more chronic and severely disabled patients were included in the MTP and patients with 21–25 headaches days per month showed the most favorable outcome. In addition to headache frequency and headache diagnosis, adherence to treatment recommendations had a significant impact on headache frequency. Adherence to more than five lifestyle recommendations was associated with a significant reduction in headache frequency. Lifestyle recommendations are obviously an important element of the behavioral treatment concept. The co-occurrence of migraine and TTH was a negative predictor regarding the reduction of headache days.

In recent years, different multidisciplinary treatment programs were established, and their effectiveness was evaluated. Harpole et al. [13] performed a prospective analysis of MTP for patients with migraine, TTH, MOH, cluster headache, and other headache entities involving headache specialists, psychologists and primary care physicians. They observed a reduction of 21.2 points in MIDAS after 6 months, including a reduction of 14.5 headache days on average within 3 months. The percentage of patients with a reduction of at least 50% in headache frequency was not calculated [13]. An MTP consisting of repeated appointments with a headache specialist, physical therapists, psychologists and headache nurses for patients with migraine, TTH, cluster headache, MOH and posttraumatic headache was analyzed prospectively by Zeeberg et al. [15] in Denmark. A significant reduction in headache days was observed in all diagnostic groups except for posttraumatic headache after a mean period of 7.8 months. Lang et al. [16] compared the effectiveness of standard headache treatment in primary health care with two intervention groups: the first group received standard treatment from specially trained neurologists in private practice; the second group was treated in a 20-h outpatient MTP at the University Pain Center. The multidisciplinary program did not outperform conventional primary care in the 6-month follow-up [16]. Later on, the same researchers compared the results to a 96-h MTP. At follow-up (after 22 weeks), 60% of the TTH patients and ~58% of the patients suffering from migraine achieved ≥50% reduction in headache days (estimated from figures in the publication). The more favorable outcome compared to our data may be a result of the 2.7 times longer intervention and the shorter follow-up period (5.5 vs. 12–18 months) [17]. Jensen et al. [9] report outcome data of MOH in a multidisciplinary headache center. At admission, the patients suffered from 27.6 headache days/month, at time of follow-up 14 headache days/month were reported. Magnusson et al. [18] compared pharmacological therapy to a multidisciplinary treatment program for patients with transformed migraine and MOH in a controlled prospective study. Several treatment approaches (for example self-management group, relaxation group, exercise group) were offered to the patients. After a mean observation period of 11.6 weeks (2–22), 35% of the patients achieved a reduction of ≥25% in headache days per month. The headache intensity was decreased by 25% in 23% of the patients. Summarizing the published data, there is a lack of well-designed studies comparing conventional treatment with MTP. However, available data suggest that an MTP may improve headache care, while efficacy may depend on an adequate duration of these programs. MTP of 20 or less hours may not be long enough. The Essen MTP for headache patients has duration of 36 h in 5 days. A 36-h program can be offered during 1 week, reducing costs compared to longer treatment programs or inpatient treatment.

The reduction of headache days after MTP is clinically relevant. Only five patients showed a relapse of MOH (11.2%) compared to relapse rates between 30 and 41% after outpatient withdrawal [5–8]. Overuse of acute medication is responsible for the development or maintenance of chronic daily headache (CDH) and successful detoxification results in a 70% return to an episodic pattern of migraine [19]. This supports our psychological and educational concept focusing on MOH, which showed efficacy in a recently published randomized trial in behavioral management sessions as well as in so-called bibliotherapy [20].

Our therapeutic concept focuses on self-efficacy and implementation of the treatment recommendations in daily life. This includes motivating patients to continue aerobic endurance sports and PMR at home. Therefore, our results indicate that higher adherence to a combination of behavioral recommendations and implementation of aerobic endurance sports and PMR is the most effective treatment. The prophylactic effect of relaxation training in headache treatment is already proven in several studies [21–23]. We also observed effectiveness of aerobic endurance sports even though the level of evidence coming from clinical trials is still weak [24, 25]. Adherence to PMR and aerobic endurance sports was better than to medical prophylaxis. Adherence to prophylactic treatment is a known problem in headache care, and factors influencing adherence to treatment in headache prophylaxis are still unclear and under debate [11, 12]. The unsatisfying adherence to medical treatment is a result of side effects and the general wish of headache patients ‘not to take a permanent medication,’ which was also found in 40% of the patients in other tertiary headache centers [26]. Thirty-five percent of our patients followed the recommendation to take prophylactic medication. This result is poorer than that achieved by Zeeberg et al. [15], whose patients show higher adherence. Thirty-nine percent of the non-MOH patients and even in the group of MOH patients took their prescribed prophylactic medication. This may be due to the shorter period of follow-up of 7.8 months compared to our period of 12–18 months. The non-adherence of our patients was explored in more detail. Main reasons for non-adherence to pharmacological prophylaxis were ineffectiveness and side effects. In addition, anxiety of side effects, the denial of daily medication intake, and general non-compliance were reasons for non-adherence. General non-compliance was reflected in only sporadic intake and in doubts based on the original indications for the drugs (e.g., depression, hypertension or epilepsy). This was surprising because patients were given information on pharmacological prophylaxis. Increasing adherence may result in improved clinical outcome even though the effects from different studies were inconsistent. Increasing the effectiveness of adherence might have more substantial impact on clinical outcome than improvement in specific medical treatments [27]. Non-adherence to treatment recommendations becomes apparent in not filling the initial prescription in 11%, termination of prophylactic regimen in 25–50%, and disregard of lifestyle modifications in 22–85% [11]. However, interventional studies on adherence in headache patients are missing.

We conclude that there is still a need to improve adherence to pharmacological recommendations by informing patients in more detail, in particular about adverse events and how to handle them. Our impression is that in the long run, behavioral modifications may be more useful than medical prophylaxis. Our patients reported that learning about headache etiology, pathophysiology, and non-pharmacological coping strategies as well as getting in contact with other headache patients (to share experiences and coping strategies and to be not left alone with the disease) were important. Even patients without reduction in headache frequency reported that visiting our MTP reduced their burden of disease because they learned how to handle headache in a more effective way. This may lead to reduction in medication intake days and lower headache-related disability as a consequence.

A methodological strength of our study is the prospective design and the long follow-up (12–18 months). This is important for testing sustained effects and may help distinguish between short-time effects (which were measured by most of the other studies) and long-lasting results and sustained changes in behavior.

The major limitation is a lack of control condition, e.g., simple advice, which was shown as effective in therapy of MOH in a population-based study by Grande et al. Notably, only two of 109, respectively, 18 of 80 patients used prophylactic medication [28]. Boe et al. [29] compared therapy of MOH patients by primary care physicians and neurologists with interest in headache and found no differences in treatment outcome. However, the patients in these two studies cannot be compared to patients treated in a tertiary headache center since these patients may have a higher burden of disease as well as several unsuccessful treatments before. Future studies should focus on treatment in MTP or rehabilitation therapy should use controlled designs, even though blinding is not possible. Also a setting comparing pharmacological and non-pharmacological prophylaxis was not possible as our concept of MTP focuses on the multidisciplinary approach to provide best care to all patients.

Some other limitations of the study must be mentioned. This was a non-randomized, open study. Patient selection led to the typical bias of a tertiary headache center taking care of severely affected and chronic headache patients. Selection criteria for admission to MTP may lead to another bias. However, this cannot be influenced by the authors. It has to be assumed that these patients are highly motivated. All our patients were classified according ICHD-II. Unlike Zeeberg et al. [15], we did not use prospective diaries in this study. However, future studies focusing on this topic should use prospective headache diaries and should include assessments for measurement of psychiatric comorbidity (especially depression and anxiety disorder) as these diseases may influence headache frequency as well as adherence to treatment recommendations.

## Conclusion

The MTP seems to be an effective approach in the treatment of frequent migraine, TTH, and MOH. However, a prospective, controlled randomized trial comparing standard treatment to MTP as part of managed care is needed for producing a real proof of effectiveness. Adherence to non-pharmacological and behavioral treatment recommendations was associated with a better outcome. Therefore, long-term treatment should focus on adherence to therapy as well as on medical information about headache diagnosis and treatment.
